# Immune checkpoint inhibition: a future guided by radiology

**DOI:** 10.1259/bjr.20220565

**Published:** 2023-02-27

**Authors:** Faraaz Khan, Keaton Jones, Paul Lyon

**Affiliations:** 1 Foundation Doctor, Buckinghamshire Hospitals NHS Trust, Amersham, Buckinghamshire, United Kingdom; 2 Academic Clinical Lecturer Nuffield Department of Surgical Sciences University of Oxford, Wellington Square, Oxford, United Kingdom; 3 Consultant Radiologist, Department of Radiology, Oxford University Hospitals, Headington, Oxford, United Kingdom

## Abstract

The limitation of the function of antitumour immune cells is a common hallmark of cancers that enables their survival. As such, the potential of immune checkpoint inhibition (ICI) acts as a paradigm shift in the treatment of a range of cancers but has not yet been fully capitalised. Combining minimally and non-invasive locoregional therapies offered by radiologists with ICI is now an active field of research with the aim of furthering therapeutic capabilities in medical oncology. In parallel to this impending advancement, the “imaging toolbox” available to radiologists is also growing, enabling more refined tumour characterisation as well as greater accuracy in evaluating responses to therapy. Options range from metabolite labelling to cellular localisation to immune checkpoint screening. It is foreseeable that these novel imaging techniques will be integrated into personalised treatment algorithms. This growth in the field must include updating the current standardised imaging criteria to ensure they are fit for purpose. Such criteria is crucial to both appropriately guide clinical decision-making regarding next steps of treatment, but also provide reliable prognosis. Quantitative approaches to these novel imaging techniques are also already being investigated to further optimise personalised therapeutic decision-making. The therapeutic potential of specific ICIs and locoregional therapies could be determined before administration thus limiting unnecessary side-effects whilst maintaining efficacy. Several radiological aspects of oncological care are advancing simultaneously. Therefore, it is essential that each development is assessed for clinical use and optimised to ensure the best treatment decisions are being offered to the patient. In this review, we discuss state of the art advances in novel functional imaging techniques in the field of immuno-oncology both pre-clinically and clinically.

## Background

Cancer immunotherapy has been revolutionised through the introduction of immune checkpoint inhibitors (ICIs). By “reactivating” intratumoral antitumour T cells, there is potential for significant improvement in patient outcomes. Such outcomes can be further amplified by harnessing the potential for diagnostic and interventional radiology in the patient pathway. Diagnostic radiology has been integrated with immunotherapies for the purposes of diagnosis, screening and therapeutic response evaluation. The importance of accurate response assessment of imaging cannot be understated with scan interpretation holding a pivotal role in the decision-making process of a patient’s oncological treatment. Image interpretation can both prevent a patient being continued on a therapy course that has little benefit as well as effective therapies being stopped prematurely. This will become more vital in exploring interventional radiology as one of the next steps towards more personalised ICI therapy. These steps can be seen as the continued realisation of the synergistic relationship between immunology and radiology first recognised as early as 1974.^1^

Interventional radiology (specifically interventional oncology) offers a range of locoregional therapies (LRTs) ranging from stereotactic ablative radiotherapy, transarterial chemoembolisation (TACE), brachytherapy (internal radiotherapy), thermal and cryoablation, irreversible electroporation and various therapeutic ultrasound bioeffects (including ablative high intensity focused ultrasound, hyperthermia, histotripsy and cavitation).^2^ The synergy between these interventions and ICI therapy promises improved patient outcomes in a variety of tumour types, following encouraging results reported in both animal models and preliminary clinical studies.^3–8^ The mechanisms underlying the complex interactions between these two modes of therapy have not been fully elucidated.^2^ However, one of the greatest theoretical advantages of combination ICI therapy is the enhancement of the innate immune system and consequent “abscopal effects.” In brief, treatment of a tumour at one site can lead to a reduction of systemic tumour burden. This is likely a result of activating antitumour specific cells via a variety of mechanisms and ICI may potentiate this systemic antitumour response. Considering the large number of permutations of interventional and ICI therapies, the optimal combination ICI therapies for specific patient and tumour characteristics have not yet been determined.

**Figure 1.**
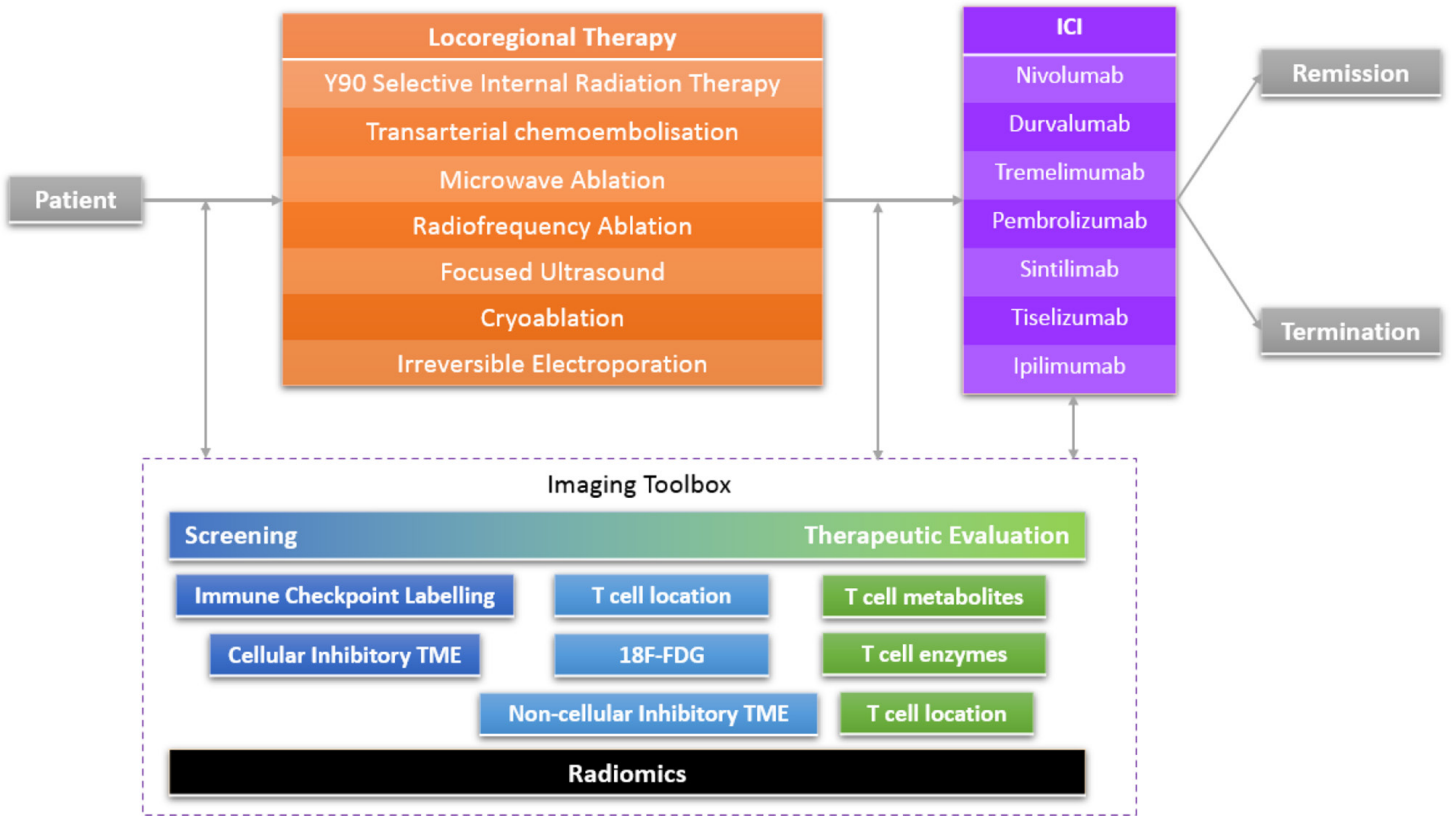
The imaging toolbox in a combined therapy pathway. ICI, immune checkpoint inhibition.

Trials to determine the optimal combination ICI therapies for specific tumour subtypes, including dose and timing are already underway, and a recent White Paper has outlined four key aims for the field of immunotherapy and interventional radiology. One of these aims includes the development and use of standard immune response criteria in imaging.^9^  Standardised imaging criteria have previously been developed prior to routine use of ICI therapies, however, further adaptations will likely be required for this new therapeutic avenue. Novel developments in diagnostic radiology will provide methods to establish the optimal LRT for specific conditions as well as implement more advanced standardised methods for evaluating response. Such information can then better guide subsequent clinical decision-making regarding the selection, scheduling, change or termination of combined therapy. Advances in radiological practice in this context are essential as the advent of liquid biopsy will inevitably lead to a greater volume of imaging to screen for cancer earlier and evaluate therapeutic response.^10,11^  This article first provides a background for a range of LRTs which is followed by a review of the new imaging modalities that could benefit the choice and therapeutic evaluation of combined locoregional intervention and ICI oncological therapy. Such technologies if implemented effectively have the potential to make significant progress in the advancement of personalised medicine (Figure 1).

## Locoregional therapies: a brief introduction

The potential of ICIs was realised in 2010 when the first Phase III clinical trial of ipilimumab, a CTLA-4 inhibitor, was reported. Advanced melanoma patients treated with a combination of ipilimumab and the peptide vaccine, gp100, benefitted from an increase in median overall survival from 6.4 to 10.0 months, compared to those treated with gp100 alone.^12^ Since then, other ICIs have been clinically approved such as pembrolizumab, a PD-1 inhibitor, for non-small cell lung cancer (NSCLC).^13^ While ICI monotherapy can benefit patients through their ability to “remove the brakes” to facilitate the activation of antitumour-specific T cells, it is rarely curative for the majority of cancer types. Systemic monoimmunotherapy can provide objective response rates as high as 45% as for advanced melanoma and 40% for colorectal cancer but lower response rates are observed for lung cancer (26%), breast cancer (24%) and hepatocellular carcinoma (HCC) (17%).^14–24^ As such, the efficacy of using more than one immunotherapy has been assessed in more recent clinical trials. A combination of ICI therapies significantly improve median overall survival in cases of advanced melanoma (39.1 *vs* 18.5 months), HCC (18.7 *vs* 11.7 months) and lung cancer (17.1 *vs* 15.7 months) compared to monotherapy.^25–28^ However, such strategies while conferring greater therapeutic benefit, increase the toxicity and consequent risk of adverse events.^29,30^ Combining ICI with LRTs may limit side-effects while improving patient outcomes.

Brachytherapy is the delivery of internal (localised) radiotherapy, often through use of wires, seeds or other implanted devices. Of note, more recent advances have led to the targeted delivery of radioactive particles via the blood supply to the tumour. This includes the use of Yttrium-90 (Y90) transarterial radioembolisation (TARE), typically used in the treatment of HCC.^31^ Y90 loaded on microspheres is delivered to a tumour via the feeding arteries. At this site, Y90 β radiation causes damage with limited injury to the surrounding normal parenchyma.^32,33^ Notably, Y90 therapy has demonstrated non-inferiority to other cancer treatments such as sorafenib, providing equal overall survival rates and decreased adverse events in locally advanced HCC.^34^

Percutaneous ablation involves the insertion of needle-like probes directly into the tumour to deliver high-energy heat (thermal ablation) or cold (cryoablation) exposure, causing coagulative necrosis of the tumour.^35^ Thermal ablation can be delivered as radiofrequency ablation (RFA) or microwave ablation (MWA). The efficacy of RFA, measured by recurrence-free survival has been found to be comparable to surgical resection in the treatment of HCC patients with early stage disease.^36,37^ Interestingly, while cryoablation and RFA provide similar efficacy in local HCC progression, cryoablation may be less utilised in the future for liver ablations due to its association with increased risk for complications including “cryo-shock,” renal impairment and thrombocytopaenia.^36,38,39^ In contrast, cryoablation has an emerging role in renal cell carcinoma (RCC) having been found to be non-inferior to MWA for adverse effects and long-term outcomes.^40–42^

Another recent study has demonstrated comparable overall survival and disease-free survival for percutaneous MWA and surgical resection of colorectal liver metastases for small resectable lesions <3cm.^43^ Irreversible electroporation (IRE) is non-thermal ablative intervention that generates external electric fields to form hydrophilic pores in cell membranes without damaging surrounding vital structures such as the vasculature or ducts. IRE is now being explored clinically for treatment of tumours such as unresectable pancreatic cancer given minimal off-target effects and the benefit of sparing vital structures such as the coeliac trunk.^44^ While IRE alone has increased survival in unresectable pancreatic cancer from 6 to 14 months, combination with immunotherapy may further improve patients outcomes. Murine and human clinical studies evaluating IRE have indeed found increased intratumoral immune response as well as overall survival rates, particularly when combined with immunotherapy.^45–47^

In contrast, focused ultrasound is a form of non-invasive targeted tumour therapy which can be used for a variety of bioeffects including high intensity focused ultrasound (HIFU), consequent with thermal ablation, subablative hyperthermia and cavitation. HIFU waves targeted to a specific location can cause tissue damage via two broad mechanisms: thermal and mechanical. Broadly speaking, thermal tissue damage shares a linear relationship to exposure time and an exponential relationship to temperature rise (typically over 60℃ causing near-instantaneous cell death). Mechanical damage is exerted by a combination of radiation force and the formation of cavitation bubbles that can either undergo vigorous oscillations resulting in shockwaves forming upon implosion or rapid movements causing shear forces on the surrounding tissue.^48,49^ Clinical applications of focused ultrasound, including HIFU are being trialled with promising results with several clinical approvals already in place including ablation of symptomatic uterine fibroids. HIFU has been demonstrated to confer very high survival rates for the treatment of contained prostate cancer treament.^50^ HIFU has limited success for high risk prostate cancer patients and in low- to intermediate risk patients may confer improved urinary continence but greater treatment failure rate in comparison to laproscopic radical prostatectomy.^51,52^ Focused ultrasound’s versatility is highlighted by its ability to enhance drug delivery non-destructively. At lower powers, focused ultrasound can also induce localised, subablative intratumoral hyperthermia and this bioeffect has been under clinical investigation for drug delivery in liver tumours.^53^ Focused ultrasound mediated cavitation can drive higher concentrations of macromolecular drugs, including immunotherapies, into solid tumours.^54,55^

TACE is currently considered the standard treatment for patients with intermediate stage HCC based on the Barcelona Clinic Liver Cancer scoring system (BCLC).^56^ There are two types of TACE, conventional (cTACE) and the novel drug-eluting beads (DEB-TACE). In cTACE a cytotoxic drug, commonly doxorubicin, emulsified in a radio-opaque contrast agent called lipiodol, is delivered to the hepatic arterial system via an intra-arterial catheter injection near to the tumour site. This results in downstream ischaemic necrosis by both cytotoxicity and ischaemia by compromising the tumour vasculature. Multiple trials and reviews have found TACE can increase overall survival when compared to conservative management of pain and complications, for unresectable HCC culminating in its incorporation into clinical guidance.^57–59^ DEB-TACE in contrast involves the delivery of beads to the tumour that both embolise the microvasculature of the tumour and simultaneously release a cytotoxic drug at a sustained rate over an extended period of time. In theory, this approach provides increased tumour and decreased systemic concentrations of the cytotoxic drug, however, clinical results are conflicting and therapeutic benefit is likely dependent on tumour staging.^60,61^

## Unlocking locoregional therapeutic potential

In 1953, it was observed that targeted external radiotherapy of one tumour area appeared to result in an antitumour response in distant areas due to stimulatory effects on the immune system.^62^ This “abscopal” effect is a shared phenomenon of LRTs. LRTs broadly promote an inflammatory microenvironment, however, there is a disparity in the immunogenicity of each ablative technique. This difference may partially account for the varying responses observed in patients treated with the same technique. It has been suggested that RFA can have pro-oncogenic effects such as greater IL-10 and TGF-b response post-treatment, but the exact mechanisms are unclear.^63,64^ Upon comparison non-ablative interventions such as HIFU, IRE, MWA and cryoablation more reliably generate a proinflammatory response which results in antitumour immunity.^65–67^ Post-Y90 therapy, increases in proinflammatory markers such as IL-1 and oxidative markers such as malondialdehyde have been observed.^68–70^ Similarly, cryoablation likely generates a greater inflammatory response than RFA as evidenced through greater IL-1b, IL-6 and TNF-a responses in rat models.^71,72^ Clinically, MWA and cryoablation have recently been found to be at least as efficacious if not superior to RFA in the treatment of HCC and liver metastases.^73,74^ Tumour-specific T cell responses have been observed in patients treated with MWA, which are associated with longer progression-free survival and remission.^75^ It has been postulated that these interventions generate an *in situ* tumour vaccine by creating a pool of tumour-associated antigens.^76–79^ Furthermore, non-ablative techniques have a higher likelihood of preserving tissue structure and neoantigens for immune cells to infiltrate and target whilst causing sufficient damage to the tumour. In keeping with this theory, T cells from patients treated with cryoablation exhibited a polyclonal shift indicating a greater variety of antigens are being recognised, likely due to the exposure of multiple tumour antigens following ablation.^80^ Correspondingly, a clinical HIFU study in breast cancer found that ablation of breast lesions lead to increases in both total immune cell populations as well as activated cytotoxic T cells in the axillary lymph nodes.^8^ However, it remains that the heterogeneous nature of the application of each LRT and cancer type complicates the decision as to which therapy is best for each patient.

These effects culminate in the conversion of the tumour microenvironment (TME) to become proinflammatory and facilitate immune cell infiltration. T regulatory cells (Tregs) are typically raised in HCC patients compared to healthy individuals, however, this is significantly reduced post-TACE.^81^ This extends to other tumour types as increases in the peripheral blood CD8:Treg ratio are observed post thermal ablation of lung, leiomyosarcoma, desmoid, liver and bone tumours.^82^ Similarly, myeloid-derived suppressor cells (MDSCs) which are associated with worse prognosis in HCC are decreased post-RFA.^83^ However, the ability to generate a proinflammatory TME and change the immune system landscape is inconsistent. Contrasting studies have found TACE to not significantly increase TAA-specific CD8 T cell populations. In addition, a recovery of anti-inflammatory cells and loss antitumour functions in the long-term post-treatment have been observed.^83,84^ Conversely, pro-tumourigenic effects have also been observed with use of some LRTs. Pro-oncogenic factors such as hypoxia inducible factor 1 a and vascular endothelial growth factor (VEGF) are increased post-RFA and TARE.^85,86^ Nevertheless, it is likely that LRTs that promote antitumour immunity would benefit from augmentation by ICI therapy and these responses are tumour-specific.^2^

Multiple case reports and a handful of trials of combination ICI and LRT therapy have been already performed (Appendix) with others ongoing. Of those already published, no life-threatening toxicities have been reported.^87–89^ In one study combining TACE with tremelimumab, authors identified an increase in the accumulation of intratumoural CD8+ T cells in all patients.^87^ However, it is not possible to determine the contribution of TACE in this context as previous trials with tremelimumab have been performed in these patients with a better baseline and prognosis.^90^ In contrast, one study did not find any increase in lymphocytic tumour infiltration using combination ICI and Y90 radioembolisation therapy.^88,89^ Interestingly, ablation post anti-PD-1 therapy in patients with stable disease or atypical responses, led to improved objective response rates and median survival.^88,89^ As these trials are limited in number and sample size, current and further larger randomised trials comparing ICI with and without LRT will help establish optimal therapies.

Supplementary Material 1.

While outcomes from these trials can be simplified to patient survival metrics it is crucial to recognise the patterns of response to therapy, most often facilitated through imaging, as ultimately this will inform clinicians if therapies are efficacious or futile for a particular patient. To effectively evaluate imaging across trials and practice, standardised criteria have been developed and modified over the last 50 years. Standardised criteria may need to be further tailored for this new therapeutic avenue to not only compare outcomes between trials but also guide clinical decision-making regarding continuation, switching or cessation of therapies.

## Standardised response criteria: a brief history



**Figure 2.**
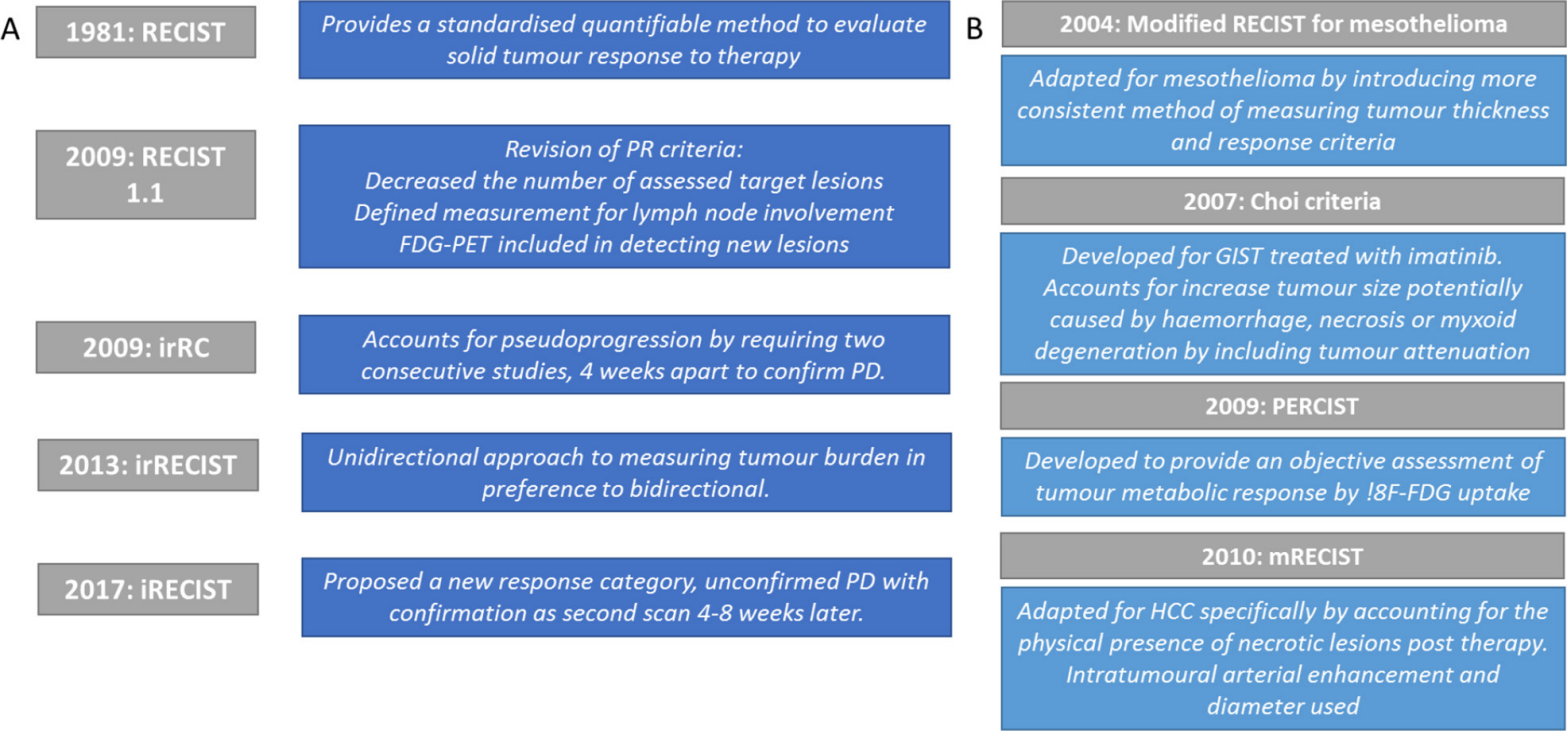
(A) The development of RECIST and key changes. (B) Cancer or modality-specific imaging criteria. irRC, immune-related response criteria; RECIST, Response Evaluation Criteria in Solid Tumours.

Imaging has been used extensively to assess response to cancer therapy. The most commonly used criteria is the “Response Evaluation Criteria in Solid Tumours” (RECIST) first published by the World Health Organisation (WHO) in their 1979 WHO handbook and in 1981 by Miller et al and at the turn of the century, formalised by the RECIST Working Group.^91,92^ This criteria provided a standardised and quantifiable method to evaluate the response of a solid tumour to therapy using imaging based on the longest measurable dimension of tumours.^91^ In modern clinical practice, CT is preferred imaging modality for RECIST and provides the workhorse, although MRI, PET and plain film are also applicable. . Responses are categorised as complete response (CR), partial response (PR), no change (NC) or progressive disease (PD). Several iterations of RECIST have since been developed with arguably the most important change being immune-related response criteria (irRC). RECIST 1.1 introduced a new method to determine if lymph nodes were pathological as well as providing new methods to measure disease progression.^93^ However, the concept of maximum tumour diameter integral to RECIST was not developed with immunotherapies in mind. This becomes most evident with the “pseudoprogression” phenomenon which is the apparent increase in tumour burden (either as an increase in initial lesion size or number of lesions visualised radiologically) soon after immunotherapy delivery. This is in fact not due to metastatic growth or progression but rather T cell intratumoral infiltration after the ICI removes the “brakes” of the immune checkpoint. This beneficial response would otherwise be incorrectly regarded as PD according to RECIST 1.1.^94,95^ The irRC accounted for this through the requirement of two consecutive studies exhibiting progression for true PD to be defined.^96^ The development of RECIST is summarised in Figure 2 and reviewed elsewhere.^97^

Further adaptations to existing commonly used response metrics including RECIST, PERCIST and CHOI, may be needed for assessing combined locoregional and ICI therapy.^98,99^ Whilst phenomena such as pseudoprogression are not common, with rates ranging from 1.49 to 11.11%, it may become more prevalent with the uptake of combination ICI therapies.^100^ LRT may initially shrink the tumour lesion but then be more readily followed by significant lymphocytic infiltration upon ICI. This will undoubtedly be influenced by the scheduling of checkpoint inhibition and specific combinations used. RECIST must be adapted to cater for this and other eventualities to ensure unnecessary termination of therapy is avoided while not risking poor detection of true PD.

In the context of LRT response, further limitations must also be addressed. Incorporation of viable tumour measurements such as through arterial enhancement in the modified RECIST (mRECIST) for HCC, have shown superiority to older criteria that do not consider this parameter in predicting overall survival of HCC patients treated with LRTs.^101,102^ Similar modifications will likely be required across multiple tumour types to correctly evaluate the potential of locoregional and combined therapies in trials. Target lesions suitable for LRT may require development of new evaluation criteria to monitor response accurately.^103^ This includes the iRECIST guidelines that require a size threshold (>10 mm for non-nodal lesion, >15 mm for nodal lesion) which may not be met for locoregional targets.

Repeat LRT is performed and might be shown in future trials to synergistically act with ICIs to create a sustained immunological pressure to breakthrough immunoinhibitory pathways. This could be most noticeable in the setting of non-thermal ablative techniques whereby tissue architecture is preserved. However, using these metrics to evaluate therapeutic response can be complicated by previous LRT to the same tumour lesion. Furthermore, a patient who experiences significant reduction of a lesion targeted by LRT may only be classed with SD if small lesions not suitable for LRT intervention or are not treated as effectively are still included in the evaluation. These challenges are compounded by the lack of information regarding optimal timing of imaging follow up for combined therapy which may lead to inaccurate assessment of responses. Whilst observations of abscopal effects from LRTs in isolation are rare, the potential remains to misclass these phenomena as new lesions and as a result disease progression.

The immune-oncology field would benefit from the widespread implementation and standardisation of emerging novel diagnostic imaging techniques. A combination of both anatomical imaging, specific to the tumour and functional imaging specific to the immune system, would enable better differentiation between pseudoprogression and PD and guide clinical decisions regarding the patients’ treatment. The power of such information also enables researchers to identify shared characteristics between and within experimental arms in trials demonstrating benefit from specific therapies, which may be tailored to specific tumour biomarkers. Such results could be used to design more “focused” trials and take a step towards personalised therapies with new criteria for patient stratification. In addition, patient-specific combined imaging may also provide insight into the complex interactions between LRTs and ICI at a more functional level.^2^

## Novel functional imaging techniques



Few clinical trials of combined, locoregional and ICI therapy have been completed, however, there are many currently taking place (Appendix). It is interesting to note that some trials have opted to use the traditional RECIST or RECIST 1.1 criteria, perhaps due to unfamiliarity with irRC. In the context of these trials, newer functional imaging techniques can provide detailed evaluation of the tumour microenvironment (TME) before, during and after combined therapy. This will allow for more conclusions to be drawn regarding screening and therapeutic potential for individual patient and tumour characteristics. Additionally, if proven beneficial, these modalities should be formally integrated into standardised criteria to ensure widespread adoption with reproducible results. We discuss state of the art advances in such novel functional imaging techniques in the field of immuno-oncology both pre-clinically and clinically.

### Glucose labelling

Radiolabelled fludeoxyglucose (^18^F-FDG) used with positron emission tomography (PET) is a now widely adopted functional imaging radiotracer used in oncology.^104^ As a labelled glucose analogue, this imaging method capitalises on a tumour’s inherent higher metabolic state resulting in greater uptake at the tumour site and thus stronger emitted signal intensity. While typically used for detection of pathologies including cancer and infective foci, ^18^F-FDG PET-CT has also been investigated for predicting prognosis. For example, ^18^F-FDG PET-CT measurements in NSCLC have been found to be reliable predictors of efficacy and patient survival at 2 weeks and 1 month post-nivolumab initiation.^105,106^ There is evidence to suggest that tumour and immune cells compete for glucose in the microenvironment. As such, the heightened tumour metabolism depletes this resource for tumour infiltrating T cells, resulting in dampened effector function. Furthermore, this is possibly linked to other tumour suppression pathways as expression of programmed death ligand-1 (PD-L1) has been shown to promote cellular pathways that drive tumour glycolysis.^107^ Positive correlations between ^18^F-FDG and PD-1/L1 expression in lung cancer and CD8 T cell infiltration have also been demonstrated.^108,109^ However, ^18^F-FDG cannot be considered as a clinically reliable surrogate for either as correlations were too weak for statistical significance. Furthermore, the relationship between ^18^F-FDG PET-CT and PD-1/PD-L1 expression is either tumour-specific or inconsistent, with direct proportionality demonstrated in bladder cancer but inverse proportionality in oral squamous carcinoma.^110,111^

A significant limitation of ^18^F-FDG PET-CT is the inability to differentiate between pseudoprogression and PD due to its lack of tumour specificity as it is taken up by physiologically active immune as well as tumour cell.^112^ While ^18^F-FDG PET-CT has been used to detect immune activity in clinical contexts including rheumatoid arthritis, atherosclerosis and graft *vs* host disease, both cancer and immune cells utilise similar pathways for glycolysis.^113,114^ This means that early ^18^F-FDG PET-CT signals post-intervention cannot be readily attributed to immune or cancer cells locally. In addition, it is also difficult to establish if lymph node signals are in fact systemic activation in reactive lymph nodes or metastases. Interpretation of ^18^F-FDG signals should be performed in the context of the specific cancer lesion; one study on brain metastases found no significant association between ^18^F-FDG uptake and immune cell infiltration while signals in the context of melanoma may either present metabolically active lesions or immune activity.^115,116^ Furthermore, ^18^F-FDG PET-CT alone cannot always correctly classify mixed metabolic responses (where lesions within the same patient may decrease while others increase in size) due to the possibility of misinterpreting unrelated synchronous events that exhibit increased glucose metabolism.^117^ To illustrate, a NSCLC study found 21% of patients treated with pembrolizumab or nivolumab who were classified as PD by ^18^F-FDG PET-CT, were in fact pseudoprogression and another 17% had dissociated responses.^118^ Moreover, all these patients experienced clinical benefit from continued ICI. The “PET Response Criteria in Solid Tumours” (PERCIST) was the original standardised criteria for evaluating ^18^F-FDG PET-CT. In a modification that mirrored the evolution of RECIST to irRC, “immune PERCIST” (iPERCIST) was developed which accounted for pseudoprogression by requiring confirmation of progressive metabolic disease at a second PET scan 4–8 weeks later.^119^

### Immune checkpoint labelling

**Figure 3.**
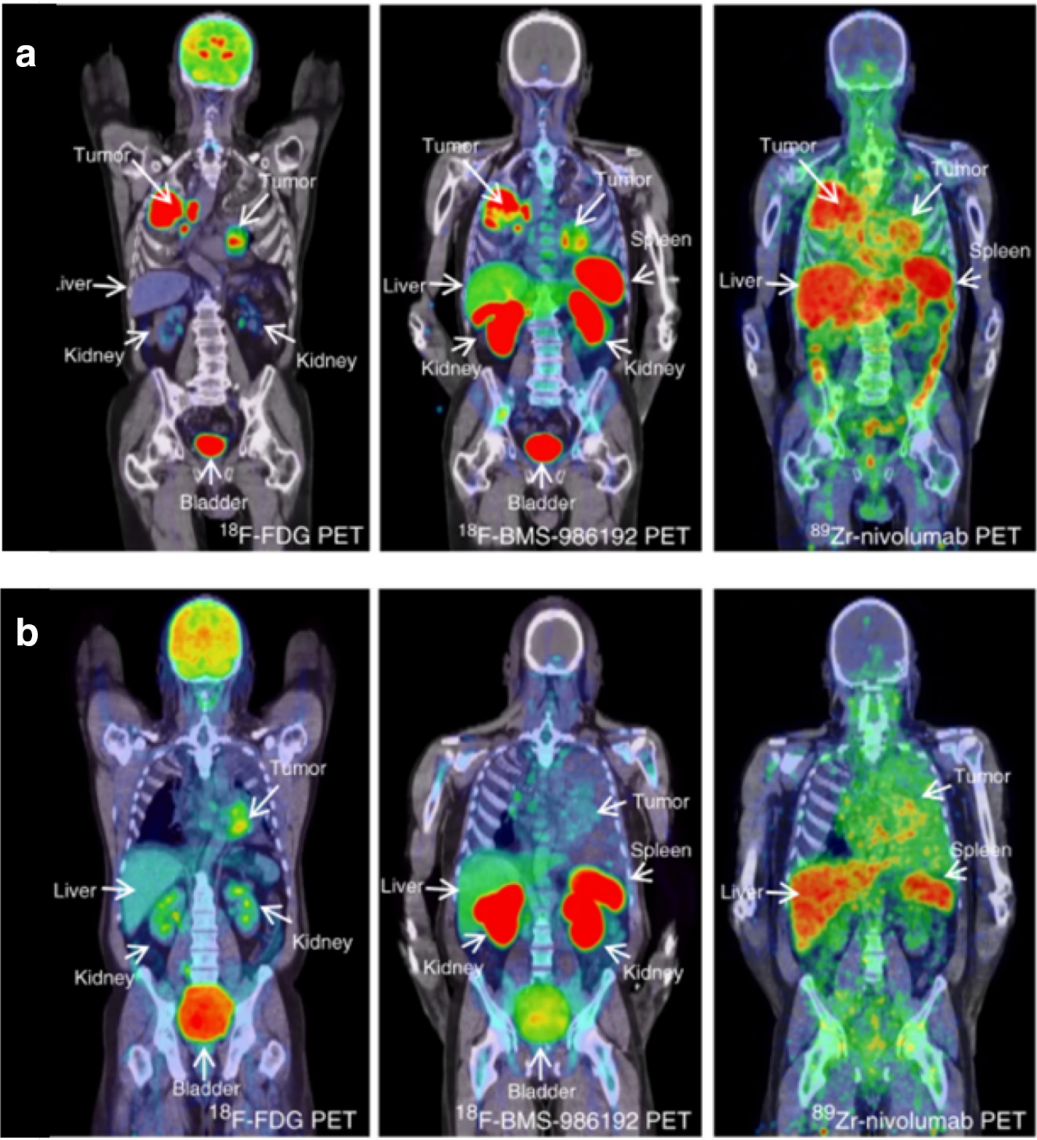
PET images of two patients, one with PD-L1 +ve (**A**) and PD-L1 -ve (**B**) tumour sites. Images demonstrating (left to right) tissue uptake of ^18^F-FDG (glucose PET tracer), ^18^F-BMS-986192 (PD-L1 PET tracer) and ^89^Zr-nivolumab (radiolabelled anti-PD1). PD-L1 +ve tumour sites demonstrate increased tracer uptake compared to PD-L1 -ve sites. Tracer uptake however is heterogeneous between and within tumour sites. Images reproduced with express permission from Nature Publishing Group under the Creative Commons License http://creativecommons.org/licenses/by/4.0/. 120 FDG, fludeoxyglucose; PD-L1, programmed death ligand-1; PET, positron emission tomography.

“Checkpoint labelling” is the direct radiological labelling of markers targeted in checkpoint inhibition through antibodies or related smaller molecules. The first in-human study for ^89^Zr-Nivolumab, was carried out in 13 advanced NSCLC patients prior to nivolumab therapy.^120^ PET uptake of ^89^Zr-nivolumab correlated with histologically confirmed PD-1 positive tumour infiltrating immune cells (*p* = 0.03) and higher peak signals were observed in therapy-responsive lesions >20 mm (*p* = 0.019), likely limited by the partial volume effect. Tumour lesion uptake was also shown to be heterogeneous between and within patients and was a stronger predictor for progression-free and overall survival compared to lab-based predictive biomarkers (Figure 3).^121^ While this labelling method appears to be clinically effective, whole antibody based imaging has its limitations including the long circulating half-lives resulting in the use of longer-lived radioisotopes and patient radiation exposure.^122^ The effective dose of ^18^F-FDG is approximately 15 mSV but 20–40 mSV for ^89^Zr-labelled antibodies.^123,124^ Moreover, the larger molecular size of antibodies (~140 kDa) may limit their penetration and uptake by tumours in comparison to the radiolabeled glucose tracer ^18^F-FDG (~180 Da), three orders of magnitude smaller.^125,126^

Smaller protein structures such as adnectins (~40 kDa) have been designed as radiotracers to bind to specific checkpoint inhibitors. In a proof of concept study, mice bearing PD-L1 -ve and +ve xenograft tumours were administered ^18^F-BMS-986192 (labelled anti- PD-L1 adnectin) systemically which specifically bound to PD-L1 +ve regions.^127^  Another anti- PD-L1 adnectin radiotracer also proved to correlate clinically with PD-L1 tumour expression and tumour lesion response to therapy.^120^  While statistically significant correlation for overall patient response to therapy as a whole was not demonstrated, the small sample size (n = 13) rather than poor radiotracer capability could account for this shortcoming. PD-L1-specific affibodies (three helical protein structures with specific protein affinity) have also been designed and tested *in vivo* in mice and rhesus monkeys and confirmed specific tracer uptake in PD-L1 positive tumours on the same day as tracer administration.^128^  This feature possibly overcomes practical limitations of whole antibody approaches which can take 5–7 days for sufficient blood clearance post-PET tracer administration. This technique conceivably further opens the avenue to precision medicine; i.e. functional imaging driving towards bespoke treatment plans based on ICI screening and monitoring.

### T cell location

**Figure 4.**
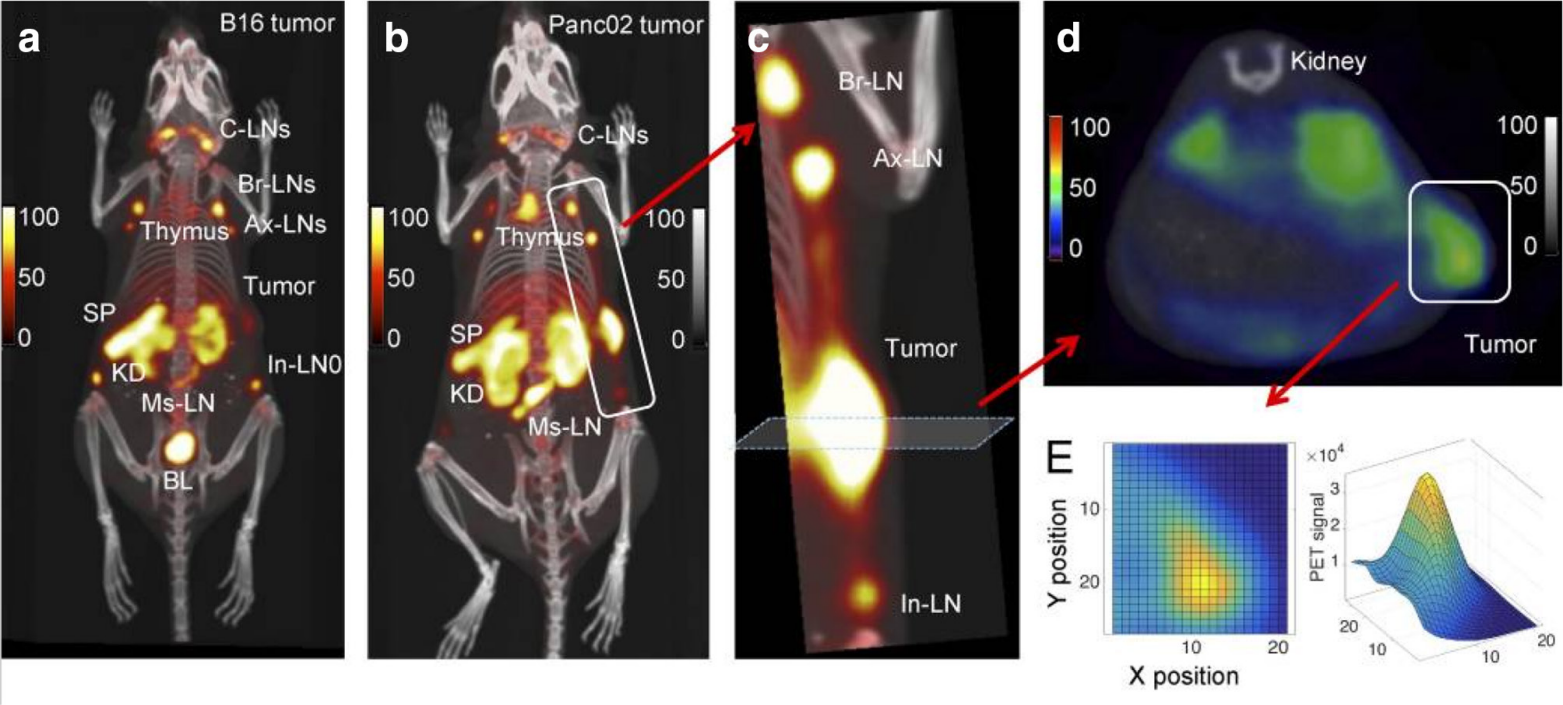
PET-CT images of mice inoculated with either B16 melanoma cells (a) or Panc02 tumour cells, a pancreatic cancer cell line (b), having administered ^89^Zr-PEGylated VHH-X118 (CD8 minibody). Images demonstrate the presence of intratumoral CD8 T cells in the tumour sites of both mice. (c) Enlarged view of the tumour site and lymph nodes from the Panc02 inocluated mouse. (d, e) Cross-section of the tumour site with CD8 signal shown (d) with chemoembolisation and 3D representation (e). CD8 T cell rich region is clearly shown within a region of the tumour.129  Images reproduced with express permission from the *Journal of Experimental Medicine*. PET, positron emission tomography.

**Figure 5.**
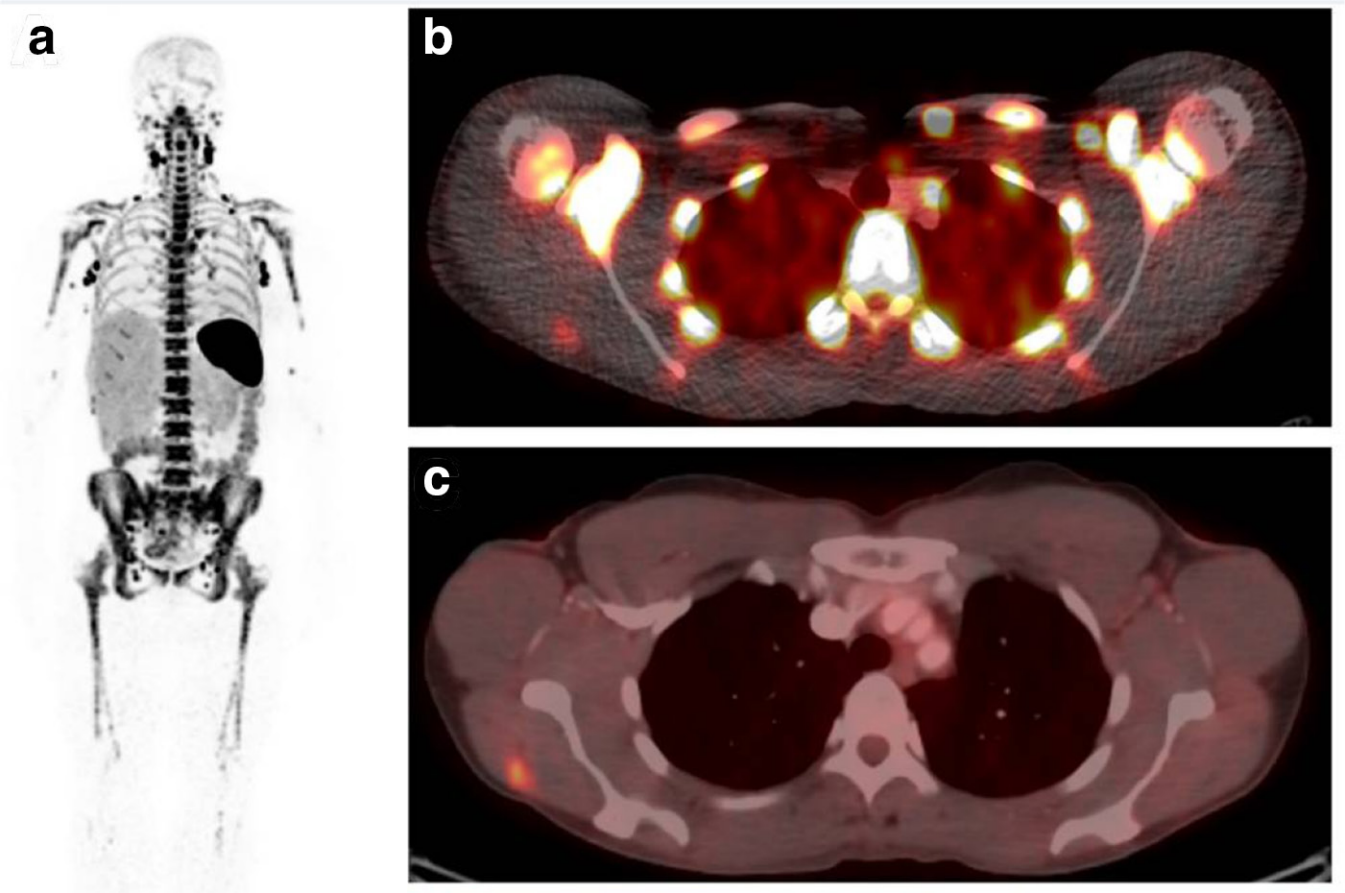
(a) Coronal projection of whole-body imaging of a melanoma patient at 24 h post-administration of ^89^Zr-IAB22M2C (CD8 minibody). Image demonstrates higher rates of uptake in the lymph nodes. (b, c) Axial fusion images of ^18^F-FDG (b) and ^89^Zr-IAB22M2C (c) at 24 h post-tracer administration. Increased uptake noted at the deltoid lesion for both tracers. This lesion could be described as “immune rich” and a site for locoregional therapy intervention in order to generate an *in situ* tumour vaccine. Images reproduced with expressed permission from JNM on behalf of Pandit-Taskar, Neeta, et al. “First-in-humans imaging with 89Zr-Df-IAB22M2C anti-CD8 minibody in patients with solid malignancies: preliminary pharmacokinetics, biodistribution, and lesion targeting.” *Journal of Nuclear Medicine* 61.4 (2020): 512–519.130 FDG, fludeoxyglucose.

**Figure 6.**
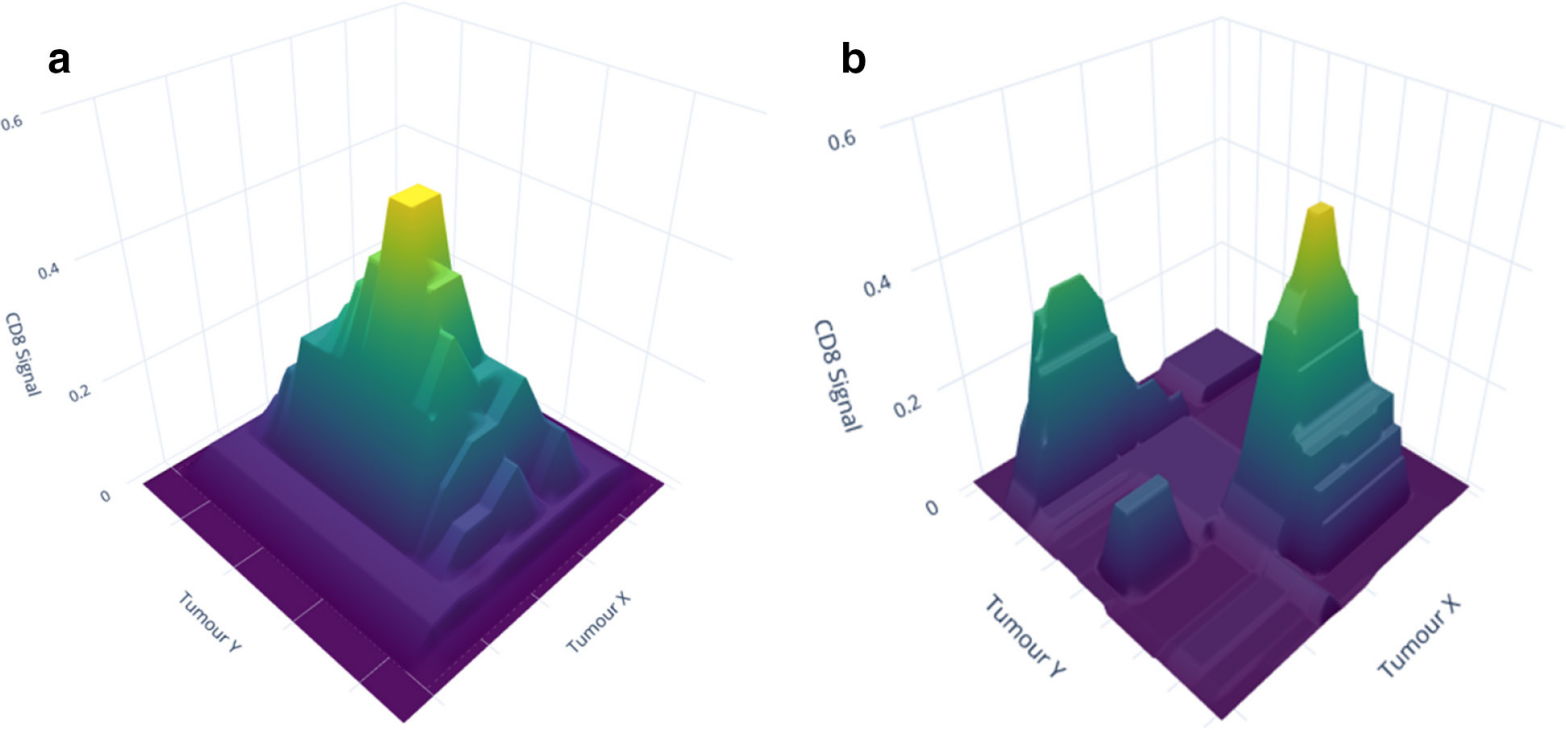
A conceptual heatmap of a tumour site demonstrating homogenous (a) and heterogenous (b) distribution of CD8 T cells within a tumour site. Hypothetical data were used to generate these heatmaps for illustrative purposes. In clinical practice, heatmaps would be generated from the PET-CT signal for each voxel of the region of interest pre- and post-combined therapy. A homogeneous distribution may suggest a better prognosis. Heterogeneous distribution may highlight key locations to be targeted by locoregional therapies to convert “immune desert” regions to “immune rich”.129 PET, positron emission tomography.

A complementary approach to imaging for combined therapy is intratumoral T-cell imaging. Whilst immune checkpoint labelling will likely have high utility in screening and stratifying patients, T cell imaging will help determine the therapeutic response and timing for ICI post-LRT. An *in vivo* mouse study demonstrated successful use of a ^89^Zr-CD8 minibody (antibody binding region fragment) for labelling T cells, generating a heatmap of PET signal within the tumour for T cell density (Figure 4). Homogeneous intratumoral T cell distribution was observed in CTLA-4 responsive B16 melanoma mice while heterogenous distribution was associated with a poor response. As such, this tool may have utility as a predictor of therapeutic response.^129^ The first in-human Phase I trial using CD8 minibodies was performed in six patients and found administration to be safe (Figure 5).^130^ Furthermore, tumour uptake occurred within 2 h of administration and in one melanoma patient, PET signal matched with pathologically confirmed intratumoral CD8 T cell infiltration. As surgical excision was only clinically indicated for the melanoma patient, it was not possible to determine if a pathological correlation existed in the HCC patient. In contrast, all four lung cancer patients were negative for ^89^Zr-CD8 minibody, however, this could be attributed to the fact that none were being treated with immunotherapy at the time of the study while the melanoma and HCC patients were. Using T-cell imaging pre- and post-LRT, it will be possible to confirm if LRT alone can induce T cell invasion to achieve “homogeneous” status. This may involve targeting T cell poor regions of the tumour specifically (Figure 6). Furthermore, these data would also guide which ICIs best enable utilisation of the *in-situ* tumour antigen pool generated through tumour cell destruction.^2^

### T cell metabolites

While the presence of intratumoral T cells may indicate therapeutic response, T cell antitumour function can be limited due to the immunosuppressive mechanisms and T cell exhaustion. To determine if LRTs and ICIs are able to “reactivate” T cells, markers of T cell activity can be evaluated. PET radiotracers of the salvage pathway of DNA synthesis have been developed, as immune cells are predisposed to use these metabolites to facilitate their heightened proliferation rate.^131^ Radiotracer clofarabine (^18^F-CFA) has been shown to be safe for human use and has increased uptake in bone marrow and lymphoid organs.^132^ When ^18^F-CFA PET was used in three glioblastoma patients being treated with immunotherapy, one patient’s intratumoral signal was elevated three-fold 3 weeks post-treatment.^133^ This corresponded with MRI confirmed immune infiltration and is a suggestive of therapeutic response. However, a decrease in signal was observed in another patient who received bevacizumab (anti-VEGF). This is possibly accounted for by the decreased perfusion, determined by advanced MRI, as a result of this therapy. It is unclear whether labelling salvage pathway metabolites will enable differentiation between pseudoprogression and true progression. Contrasting studies have identified ^18^F-CFA PET as a potential marker for HCC imaging of cancer cells due to their higher proliferation rate and similar reliance on the salvage pathway.^134,135^ It is possible this is dependent on the tumour type, therefore, further studies are required to determine the utility of these radiotracers in combined therapy for different tumour subtypes. Other studies have begun investigating the use of cytokine labelling as prognostic factor pre- and post-ICI but these are early in development with limited success to date and similarly to other types of tracers, is likely to be cancer specific.^136–138^

### T cell enzymes

A T cell specific alternative is “enzyme labelling,” including granzyme B which is expressed by activated CD8 T cells and is involved in immune mediated tumour cell killing.^139^ In a mouse model, the PET imaging agent GZB was tested for quantifying granzyme B release at CT26 and MC38 tumour sites before and after anti-PD-1 and anti-CTLA-4 therapy.^140^ GZB PET signal positively correlated with CD8 T cell subpopulations and Th1 inflammatory cytokines and negatively correlated with immunosuppressive cell types at the tumour site and anti-inflammatory cytokines at draining lymph nodes for MC38. A different approach to granzyme B imaging has also been developed where a small peptide “nanoreporter” is conjugated onto the ICI and is cleaved by granzyme B to activate a fluorescent signal which is detected non-invasively.^141^ Higher signal levels were observed in therapy-responsive tumours of treated mice compared to those with therapy-resistant tumours or not treated. Intriguingly, this signal was detected before tumour shrinkage was observed with other imaging modalities and was reported to correspond with survival rates. Other mouse model studies have also found a strong predictive ability of granzyme labelling to distinguish between responders and non-responders to ICI.^142^ Despite not yet being investigated in human trials, granzyme B appears to be a promising target for characterising the TME to assess therapeutic response.

### Cellular and non-cellular inhibition

For screening and prognostic purposes, it may be useful to characterise the inhibitory aspects of the TME as well. Anti-CD11b radiotracers have been developed to image “myeloid derived suppressor cells,” which are known to be an important negative prognostic marker for response to ICI, and have accurately characterised the TME in mouse models.^143,144^ Non-cellular aspects of the inhibitory TME such as hypoxia should also be considered and novel BOLD MRI techniques may also have a supportive diagnostic role here.^145,146^ Carbonic anhydrase (CA) contributes to the acidification of the TME under hypoxic conditions and is strongly expressed by some tumours.^147,148^ CA expression was also found to correlate with intratumoral Foxp3+ T cell density and poorer survival in NSCLC patients.^149^ Labelling of CA has already been demonstrated in renal cell cancer patients for lesion detection and as a marker for poor prognosis.^150^ However, there is no evidence to suggest whether CA is prognostic for therapeutic response to locoregional intervention or ICI. By contrast, imaging of normalising extracellular pH post-LRT has been identified as a positive prognostic factor for therapeutic response in animal models and may have a role in clinical assessments with further validation.^151^ CEST MRI may have a supporting diagnostic role in this regard.^152^

#### Tumour perfusion

Evaluating the tumour vasculature has enabled clinicians to not only determine the tumour’s ability for local invasion and metastasis but also predict response to different therapies. Greater tumour perfusion may allow for superior drug delivery and immune infiltration. In separate studies, greater blood flow on CT perfusion studies for rectal carcinoma and head and neck cancers were associated with better chemotherapy and radiotherapy responses.^153–155^ Furthermore, the immunoinhibitory limitations posed by a hypoxic environment may not be present in well-vascularised tumours. These lesions might be easier targets for combined locoregional interventions and immunotherapy as found in the use of antiangiogenic therapies. It has also been proposed that MRI perfusion studies may enable clinicians to differentiate between tumour recurrence and necrosis.^156^ If validated, incorporating this modality post-LRT in follow-up will ensure greater accuracy of response evaluations.

## Radiomics

Radiomics is a field that involves using algorithms to automatically extract and quantify features from an image. This approach has been employed to characterise tumours as inflamed or non-inflamed using the CD8 cell signature.^157^ The algorithm was trained by combining the contrast-enhanced CT scans of 135 patients with advanced solid tumours from the MOSCATO trial, with respective RNA-sequencing data of the CD8B gene. From 84 input variables, 8 variables were retained during the training step to generate the radiomic signature which included a combination of location, density of the peritumoral peripheral ring, kilovoltage peak and grey-level emphasis. The radiomic score indicated whether there was high or low abundance of CD8 infiltrate on analysis of a CT scan. The algorithm was validated with additional data sets of known CD8 T cell infiltration density. Furthermore, a higher radiomics score, indicating an inflamed tumour, was associated with objective response at 3 and 6 months post-anti-PD-1/PD-L1 therapy as well as overall survival.

As with cellular labelling, this characterisation has implications for LRT in assessing whether lesions would benefit from direct intervention to switch a tumour from non-inflamed to inflamed even before using ICI for a specific patient. Pathological studies have shown superiority of selective internal radiation (SIRT) over TACE for intratumoral effector T cell recruitment in HCC. The ability to verify T cell infiltration intra- or post-operatively and by tumour region, without need for an invasive biopsy, will lead to more informed decision-making regarding additional interventions or ICI use.^158^ Multiple models have also been developed to predict prognosis of HCC patients post TACE therapy.^159^ It has generally been found that models which incorporate both radiomics and clinical features such as α-fetoprotein and Child-Pugh score (score of severity of long-term liver disease) perform best in predicting survival.^160–162^

Radiomics is an ever-advancing field and its use has been applied clinically to other novel imaging techniques including dual energy CT, diffusion- weighted imaging (DWI), intravoxel incoherent motion DWI (IVIM-DWI), functional MRI, BOLD MRI and perfusion studies.^163–169^ It is inevitable that future directions will see increasing application of radiomics to such novel imaging techniques for the purposes of advancing diagnostic and prognostication in oncology and guiding optimal immunotherapy treatments. In-depth characterisation of the whole TME by imaging can be achieved unlike biopsies which can only sample a small fraction of the heterogeneous TME. Screening patients could be performed to ensure specific therapies are only considered for those with predicted therapeutic benefit. Stratification in this manner will likely improve patients’ outcomes and limit futile treatments, avoiding delays in appropriate treatment and eliminate unnecessary adverse effects. In addition, it may lead to greater personalisation of combined locoregional and ICI therapy. Prognostic characteristics within patient treatment groups may also be identified with more imaging modalities available. This will likely include integration of liquid biopsy data which through analysis of hypermutated ctDNA and microsatellite instability could predict which patients would best respond to ICI therapy.^170,171^ Such findings can then be used to guide future trials and clinical practice (Figure 1).

## Challenges: present and future

The therapeutic potential of combined locoregional and immunotherapy may be game changing in the development of more successful oncological treatments in the era of personalised medicine. However, we remain early in this journey and there are several broad and specific challenges. The realisation of this therapeutic pathway will rely on the success of several carefully designed pivotal clinical trials, as there are many combinations of LRTs, immunotherapies and imaging modalities. Furthermore, the relative timing of the therapies and imaging as well as the criteria to be adopted is not consistent. The heterogeneous nature of these trials across a wide range of tumour subtypes may complicate comparisons and weaken conclusions to be made for future evidence-based practice. The expense of such combination device and drug trials can be prohibitive, both in terms of setup costs of earlier phase trials to demonstrate safety and in later phase trials where large numbers of patients are likely to be required to demonstrate efficacy. A plausible solution may include the use of a modified basket or umbrella trial whereby patients are first screened by biotracers discussed in our review and then allocated to specific trial arms.^172^ In parallel, the use of case series would provide a foundation for future larger scale trials.

As with all cancer treatments, there is likely a biological limitation in some patients through the inevitable selection process of resistant cancer cells and immunologically “cold” tumours.^173^ Combination ICI therapy and LRT may go some way to addressing this, but this comes at the cost of increasing toxicity and consequent adverse effects. Further, the response evaluation of LRTs may be more challenging due to previous interventions including radiotherapy and ablation.

## Conclusion

Overall, there are multiple imaging techniques and modalities, including functional imaging, that diagnostic radiology can offer to support the field of immunotherapy. Specifically, this may be in terms of diagnosis, directing therapy and following treatment response, but furthermore in tailoring the most appropriate immunotherapy treatments and LRTs. Stepwise advances towards more and more personalised oncological treatment pathways are continuously being made. Both non-invasive and minimally invasive forms of diagnosis and treatment are being developed and implemented. The widespread adoption of liquid biopsy is approaching for screening, diagnosis and prognostication, which in combination with the novel imaging techniques discussed, will provide even more information to enable multidisciplinary teams to effectively generate patient-specific algorithms for treatment and surveillance. Different LRTs have theoretical benefits to being used in combination with ICIs thus, many trials are now taking place. Diagnostic radiology has become pivotal in the field of immunotherapy and its utility will only increase with combined ICI and interventional radiological therapies now being actively pursued. The synergy between LRTs and ICI is only now beginning to be assessed clinically and is in no way optimised. To ensure all relevant information can be extracted from these trials, existing radiological response metrics may need to be adapted and implemented. Incorporation of new imaging technologies into patient screening and therapeutic response evaluation will facilitate progress. This will enable relevant comparisons within and between trials to be made as well as provide more information for clinical decision-making regarding treatment. The future of medical oncology is pivotal on the integration of these new technologies, treatments, and expertise of the multidisciplinary team including close collaboration between oncologists and radiologists.
