# Supplementary material for: Immune checkpoint inhibition: a future guided by radiology
Source: Br J Radiol. 2023 Feb 27;96(1147):20220565. doi: 10.1259/bjr.20220565 (PMC10321249; doi:10.1259/bjr.20220565)
Supplement: Supplementary Material 1. [file bjr.20220565.suppl-01.pdf]

Table 1 Completed trials of combined locoregional and checkpoint inhibition therapies

Clinical Trial	Number of Participants	Cancer	Locoregional therapy	Checkpoint Inhibitor	Imaging Assessment
NCT04707547	5	Intrahepatic metastasis	RFA	Nivolumab	N/A
NCT01853618	61	HCC or BTC	TACE, RFA or Cryoablation	Tremelimumab	RECIST, iiRC
NCT03005002	9	Hepatic metastasis	Radioembolisation	Durvalumab, Tremelimumab	RECIST 1.1
NCT03380130	41	HCC	Y90 Radioembolisation	Nivolumab	RECIST 1.1
NCT03939975	50	HCC	RFA or MWA	Pembrolizumab, nivolumab	N/A

RFA: radiofrequency ablation, HCC: hepatocellular carcinoma, BTC: biliary tract carcinoma, TACE: transarterial chemoembolisation, RECIST: Response evaluation criteria in solid tumours, MWA: microwave ablation

Clinical Trial (Phase)	Number of Participants	Cancer	Locoregional therapy	Checkpoint Inhibitor	Imaging Assessment
NCT04518852 (P2)	60	HCC	TACE	Anti-PD1 antibody	mRECIST
NCT03572582 (P2)	49	HCC	TACE	Nivolumab	mRECIST
NCT04605731 (P1)	32	HCC	Y90 SIRT	Durvalumab, Tremelimumab	RECIST 1.1, mRECIST, imRECIST
NCT04340193 (P3)	765	HCC	TACE	Nivolumab, Ipillimumab	N/A
NCT03143270 (P1)	14	HCC	TACE	Nivolumab	RECIST 1.1
NCT03101475 (P2)	70	Liver Mets	RFA	Durvalumab, Tremelimumab	RECIST 1.1, iRECIST
NCT02837029 (P1)	27	HCC	Y90 SIRT	Nivolumab	RECIST 1.1
NCT03812562 (P1)	2	HCC	Y90 SIRT	Nivolumab	RECIST 1.1
NCT04108481 (P1/2)	18	Liver Mets	Y90 SIRT	Durvalumab	RECIST 1.1, mRECIST, iRECIST

NCT02821754 (P2)	90	HCC, BTC	TACE, RFA, Cryoablation	Durvalumab, Tremelimumab	RECIST
NCT03914352 (n/a)	40	HCC	TACE	Anti-PD1 antibody	N/A
NCT04238637 (P2)	50	BTC	Y90 SIRT	Durvalumab, Tremelimumab	RECIST 1.1
NCT02437071 (P2)	34	Liver Mets	RFA	Pembrolizumab	RECIST 1.1
NCT04652440 (P1/2)	30	HCC	RFA	Tislelizumab	N/A
NCT04297280 (P2)	25	HCC	TACE	Sintilimab	mRECIST
NCT04273100 (P2)	56	HCC	TACE	Lenvatinib	RECIST 1.1
NCT04653389	30	HCC	TACE	Sintililmab	mRECIST
NCT02913417 (P1/2)	26	Liver Mets	Y90 SIRT	Ipilimumab, Nivolumab	RECIST
NCT03753659 (P2)	30	HCC	RFA, MWA, Brachytherapy or combination with TACE	Pembrolizumab	RECIST 1.1
NCT04220944 (P1)	45	HCC	TACE, MWA	Sintililmab	mRECIST
NCT03937830 (P2)	22	HCC	TACE	Durvalumab, Tremelimumab	RECIST 1.1
NCT04174781 (P2)	61	HCC	TACE	Sintililmab	mRECIST
NCT04517227 (n/a)	30	HCC	TACE	Durvalumab	mRECIST
NCT04479527 (P2)	34	HCC	TACE	Camrelizumab	mRECIST
NCT03638141 (P2)	30	HCC	TACE	Durvalumab, Tremelimumab	mRECIST
NCT03778957 (P3)	710	HCC	TACE	Durvalumab	mRECIST
NCT04472767 (P2)	35	HCC	TACE	Nivolumab	mRECIST
NCT04124991 (P1/2)	24	HCC	Y90 SIRT	Durvalumab	mRECIST

NCT03397654 (P1/2)	26	HCC	TACE	Pembrolizumab	mRECIST
NCT03099564 (P1)	30	HCC	Y90 SIRT	Pembrolizumab	RECIST 1.1, mRECIST
NCT03033446 (P2)	40	HCC	Y90 SIRT	Nivolumab	N/A
NCT04522544 (P2)	84	HCC	Y90 SIRT	Tremelimumab. Durvalumab	RECIST 1.1
NCT04727307 (P2)	202	HCC	RFA	Atezolizumab	mRECIST
NCT04246177 (P3)	950	HCC	TACE	Pembrolizumab	RECIST 1.1, mRECIST
NCT03630640 (P2)	50	HCC	Electroporation	Nivolumab	mRECIST
NCT04541173 (P2)	128	HCC	Y90 SIRT	Atezolizumab	RECIST 1.1, mRECIST
NCT04224636 (P2)	106	HCC	TACE	Atezolizumab	RECIST 1.1, mRECIST
NCT04268888 (P2/3)	522	HCC	TACE/TAE	Nivolumab	RECIST 1.1
NCT04592029 (P1)	36	HCC	TACE	Sintilimab	RECIST 1.1, irRECIST, mRECIST

(Px): Phase x, RFA: radiofrequency ablation, TACE: transarterial chemoembolisation, MWA: microwave ablation, SIRT: selective internal radiation therapy, TAE: transarterial embolisation, HCC: hepatocellular carcinoma, BTC: biliary tract carcinoma, RECIST: Response evaluation criteria in solid tumours, irRECIST: immune related RECIST, mRECIST: modified RECIST, imRECIST: immune modified RECIST
